# River state classification combining patch-based processing and CNN

**DOI:** 10.1371/journal.pone.0243073

**Published:** 2020-12-03

**Authors:** Takahiro Oga, Ryosuke Harakawa, Sayaka Minewaki, Yo Umeki, Yoko Matsuda, Masahiro Iwahashi

**Affiliations:** 1 Department of Electrical, Electronics and Information Engineering, Nagaoka University of Technology, Nagaoka, Japan; 2 Department of Computer Science and Engineering, National Institute of Technology, Yuge College, Kamijima-cho, Ochi-gun, Ehime, Japan; 3 Department of Civil and Environmental Engineering, Nagaoka University of Technology, Nagaoka, Japan; Bristol University/Remote Sensing Solutions Inc., UNITED STATES

## Abstract

This paper proposes a method for classifying the river state (a flood risk exists or not) from river surveillance camera images by combining patch-based processing and a convolutional neural network (CNN). Although CNN needs much training data, the number of river surveillance camera images is limited because flood does not frequently occur. Also, river surveillance camera images include objects that are irrelevant to the flood risk. Therefore, the direct use of CNN may not work well for the river state classification. To overcome this limitation, this paper develops patch-based processing for adjusting CNN to the river state classification. By increasing training data via the patch segmentation of an image and selecting patches that are relevant to the river state, the adjustment of general CNNs to the river state classification becomes feasible. The proposed patch-based processing and CNN are developed independently. This yields the practical merits that any CNN can be used according to each user’s purposes, and the maintenance and improvement of each component of the whole system can be easily performed. In the experiment, river state classification is defined as the following problems using two datasets, to verify the effectiveness of the proposed method. First, river images from the public dataset called *Places* are classified to images with *Muddy* labels and images with *Clear* labels. Second, images from the river surveillance camera in Nagaoka City, Japan are classified to images captured when the government announced heavy rain or flood warning and the other images.

## Introduction

In Japan, there has been an increasing number of annual occurrences of heavy rainfall with hourly precipitation of more than 50 mm (https://www.data.jma.go.jp). Accordingly, flood damage has been increasing in many places. Therefore, we need to develop a method to prevent or reduce flood damage. To assess flood risk, it is necessary to monitor the appearance of a river.

To satisfy this requirement, methods using water level gauges have been proposed [1–3]. These methods detect floods by measuring the rise of the water level. Recently, water level gauges with ultrasonic technology [1] and those with a radar technique [2] have been used. These methods emit radio waves or ultrasonic waves and measure the time taken to return from the measured point on the water surface; from this information, the water level can be determined. With these techniques, we do not need to locate the water level gauges underwater and can avoid malfunctions caused by flowing water. However, these methods have the problem that they cannot accurately measure the water level if sediment exists on the measured points. There is also the problem that the cost of installing the equipment with large wireless communication sensors is high [4]. As another solution to monitoring river optical properties, a method using a turbidity meter has been proposed [5]. Because this method measures the muddiness underwater, the burden of installed the equipment is greater and it cannot be used when the water flow is rough. Furthermore, methods for mapping the flood inundation areas in satellite images have been proposed [6, 7]. However, these methods have high cost to capturing the satellite images with sensors.

To solve these problems, methods have been proposed for measuring the water level by capturing images of rivers [4, 8–11]. These methods have the merit that the cost of locating the equipment is low because only general surveillance cameras are needed. In fact, although water level gauges are not likely to be located in small rivers [4], surveillance cameras can be easily located in small rivers, as well as major rivers [12]. Therefore, by analyzing images obtained by a surveillance camera, it is possible to monitor the appearance of rivers without the need for special sensors such as water level gauges or turbidity meters. Specifically, a method using a surveillance camera has been proposed [13], which performs binary classification of whether a river appearance is normal or a potential flood risk, using a convolutional neural network (CNN) [14–20]. To improve the classification performance, this method performs image segmentation using a pyramid scene parsing network (PSPNet) [21], to remove irrelevant background areas in advance. To the best of our knowledge, this study [13] is the first work on classification of river appearance using a surveillance camera. However, this method has the following two problems. First, because there is a limited amount of training data when a flood risk exists, the performance may be poor. Second, because images include some areas that are irrelevant to classification, the irrelevant areas may decrease the performance.

In this paper, we propose a method for classifying the river state (a flood risk exists or not) from river surveillance camera images by combining patch-based processing and CNN. The patch-based processing is a technique for training CNN using multiple patches in an image [22]. When the number of training images for CNN is limited, the patch-based processing is beneficial for data augmentation. Specifically, we first produce more training data for flood risk by generating multiple patches from each image. In this processing, by generating multiple patches from each image, in a sliding window fashion [23], we can produce more training data for flood risk, to train the CNN. Thus, we can avoid the performance degradation that is caused by overfitting because of the small amount of training data. Second, we derive a patch selection scheme for obtaining only relevant patches. In this processing, we remove patches that are irrelevant to the assessment of flood risk; these are the patches (e.g., patches corresponding to the background area) that are not useful for the classification. Furthermore, we apply CNN to the selected patches and obtain the final classification results by majority voting [16, 24]. Specifically, the majority voting first produces classification results (of whether a flood risk exists), i.e., votes, for each patch in an image. Then, the majority voting classifies the image to the class receiving the largest number of votes. This enables the method to emphasize the effect of patches that are relevant to the flood risk, and successful classification (of whether a flood risk exists) becomes feasible.

## Aim of this work

We aim at not development of a new kind of CNN but development of pre-processing and post-processing that can be introduced into any CNN. Recently, CNN software (e.g., Microsoft Azure (https://azure.microsoft.com/ja-jp/), IBM Watson (https://www.ibm.com/watson/jp-ja/), Amazon Web Services (https://aws.amazon.com/jp/) and Google Cloud (https://cloud.google.com/products/ai)) has become widespread. A user can introduce our patch-based processing into each user’s CNN. In the system development, it is often that multiple providers develop each component of the system independently and perform system integration finally. Because our patch-based processing and CNN are developed independently, the maintenance and improvement of each component of the whole system can be easily performed. For example, if a new CNN is presented, we can classify the river state by only replacing the current CNN with the new CNN without adjustment of the cooperation of CNN and patch-based processing. Experimental results with real river surveillance camera images show that the adjustment of general CNNs [14–20] to the river state classification becomes feasible.

## Proposed pre-processing and post-processing for river state classification

**Fig 1:**
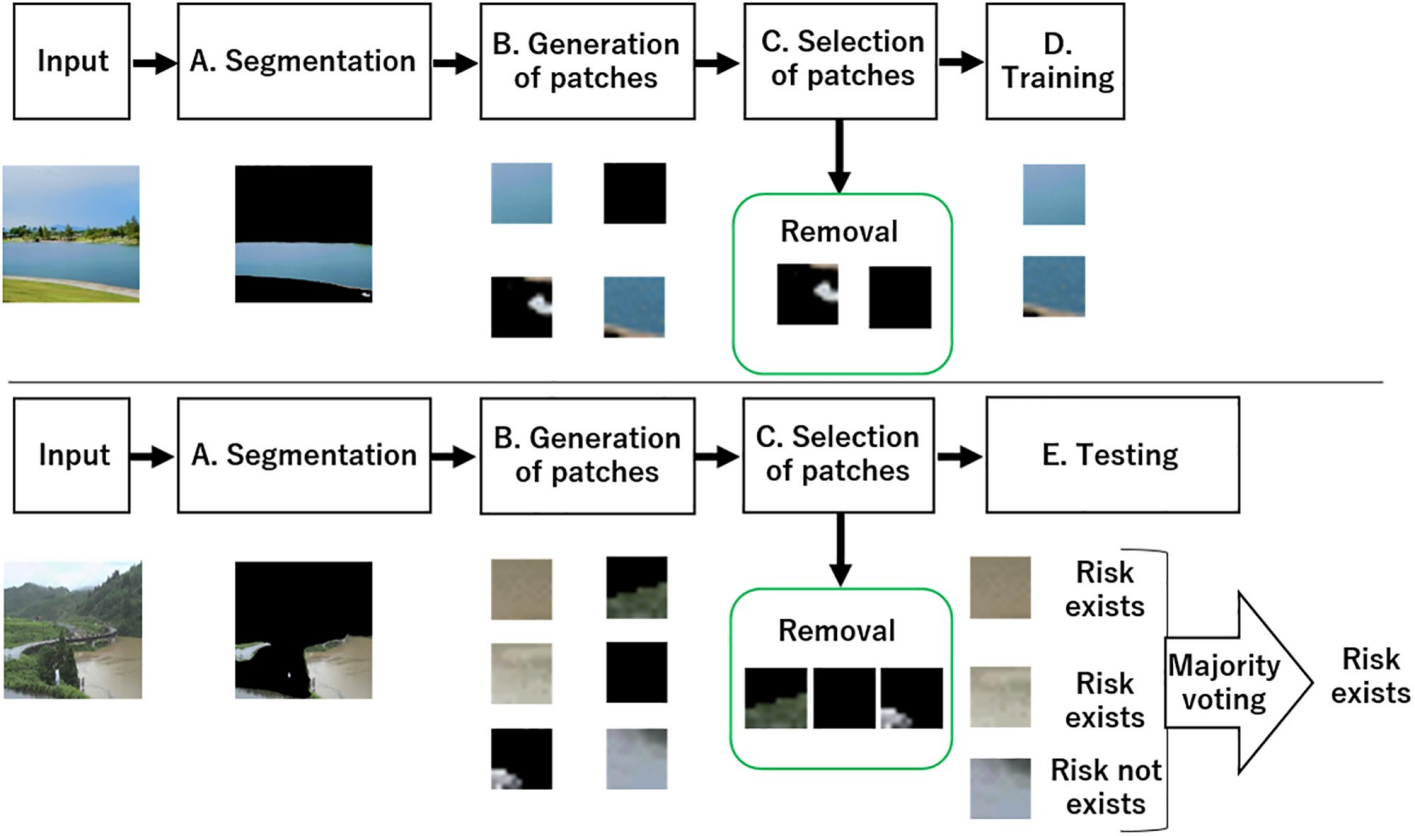
Overview of the proposed method.

Fig 1 shows an overview of the proposed method, which consists of the following five phases:

1. image segmentation for obtaining water regions;
2. patch-based data augmentation for CNN;
3. selection of patches relevant to classification;
4. training of CNN using the selected patches;
5. testing with an ensemble of multiple patches for classification.

The details of each processing phase are shown below.

### A. Image segmentation for obtaining water regions

In this process, we detect water regions in an image and perform zero padding for other regions, so that the subsequent processing can use only regions relevant to the classification. Specifically, we perform semantic segmentation based on PSPNet [21], which is currently one of the state-of-the-art methods. PSPNet achieved high performance for the ADE20K dataset [25], which consists of 150 classes (see Table 1), including classes related to water regions. In this study, we therefore trained PSPNet by using the ADE20K dataset. Because water regions may be classified as “water”, “sea”, “lake”, and “natatorium”, as well as “river”, we defined the regions classified as those classes to be water regions. In this way, we were able to successfully detect water regions from a river image.

**Table 1:** Example of classes included in the ADE20K dataset [25].

Classes related to water	Other classes	
water	grass	fence
river	tree	wall
sea	mount	road
lake	sky	stone
natatorium	flower	sand

**Fig 2:**
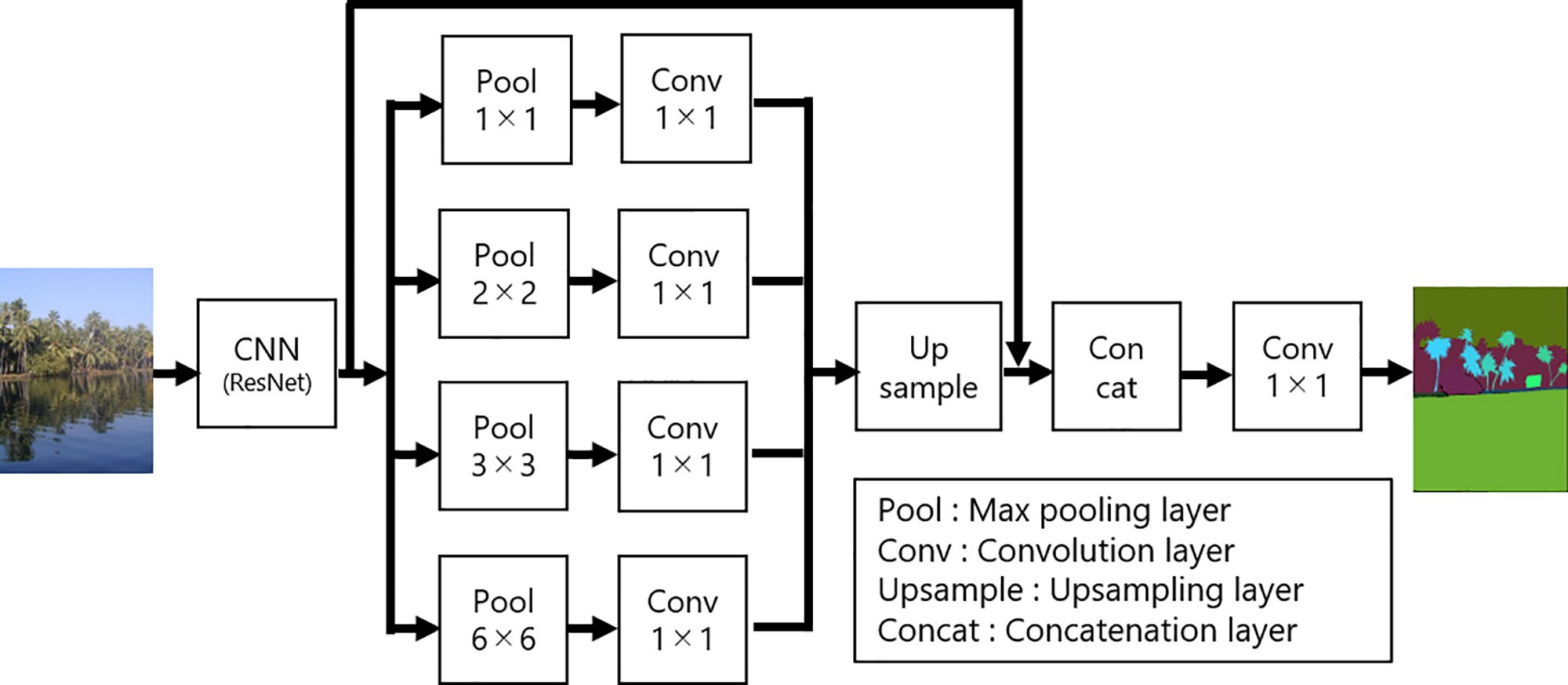
Overview of the flow in PSPNet [21].

Here, we explain the detailed structure of PSPNet. Many CNNs [14–20] consist of convolutional layers, pooling layers, and fully-connected layers. In contrast, some CNNs for semantic segmentation [21, 26, 27] replace fully-connected layers by convolutional layers and output a two-dimensional map instead of classes. Fig 2 illustrates the flow of PSPNet. First, we extract a feature map using a residual neural network (ResNet) [18], without fully-connected layers, as an encoder. We then obtain feature maps containing information with different scales by inputting a feature map to the pooling layers, which have various sizes. Through convolution of these feature maps, we can discover the global and local characteristics of an image. Furthermore, we perform upsampling to change each feature map to the size of an original feature map and concatenate the resulting maps; this produces a final feature map that possesses both global and local characteristics of an input image. Finally, we obtain the segmentation result by applying 1 × 1 convolution to the final feature map. As a result, PSPNet enables accurate segmentation of an image that includes water regions and various backgrounds.

### B. Patch-based data augmentation for CNN

In this process, we perform data augmentation for CNN, i.e., patch generation from each river image. In general, features that are necessary for classification may be lost by dividing an image into patches. However, in our river state classification, we do not need to determine the detailed texture in local areas, because the global characteristics of the water regions are crucial for predicting flood risk. Therefore, we do not need to tackle this problem; it is sufficient to simply generate multiple patches from each river image. In the experiment shown below, we generated patches of 32 pixels × 32 pixels whose slide width is 16 pixels. The number of patches generated from a single image is defined as follows:

$$\begin{array}{c} N=(\frac{I-C}{P}+1{)}^{2}, \end{array}$$ (1)

where *N* is the number of patches, *I* is the image width, *P* is the sliding window size, and *C* is the patch width, assuming that all images and patches are square. In this manner, we can increase the amount of data to compensate for insufficient of the training data for river state classification.

### C. Selection of patches relevant to classification

In this process, we select patches that are relevant to flood risk from those generated for the training of the CNN. Specifically, by removing patches with zero-padded pixels, (i.e., non-water regions), we select only the relevant patches (i.e., water regions), which are then used to suitably train the CNN. For this purpose, we need to define the criterion for removing the patches. In the proposed method, we define *Th* as the maximum fraction of pixels whose values are zero; patches with a greater proportion of zero pixels are removed. We determine a suitable criterion by changing *Th*; we explain how *Th* was set in the experiment shown below.

### D. Training of CNN using selected patches

In this process, the selected patches are used to train the CNN for the classification of the river state. Our proposal is not the design of a new CNN model but the development of a classification method that can incorporate any CNN. In the experiment, we demonstrate that the proposed method improves the performance of various types of CNNs, including AlexNet [14], NIN [15], GoogLeNet [16], VGGNet [17], ResNet [18], WideResNet [19], and ResNeXt [20].

### E. Testing with an ensemble of multiple patches for classification

Here, we describe the test phase using the trained model for river state classification. When targeting river images, we can accurately classify patches showing water regions, but the performance of classification may be worse for non-water region patches. However, the proposed method can remove the non-water region patches. Therefore, a simple majority voting strategy, as used in [24], enables the successful classification of the river state.

Specifically, we generate multiple patches from each test image in the same manner as training. We then input the resulting patches to the trained CNN model and calculate the probability that a patch belongs to a water region. By using multiple classification results per image, we obtain the final classification result by majority voting. This results in accurate classification even though several patches may have been misclassified by the CNN.

**Fig 3:**
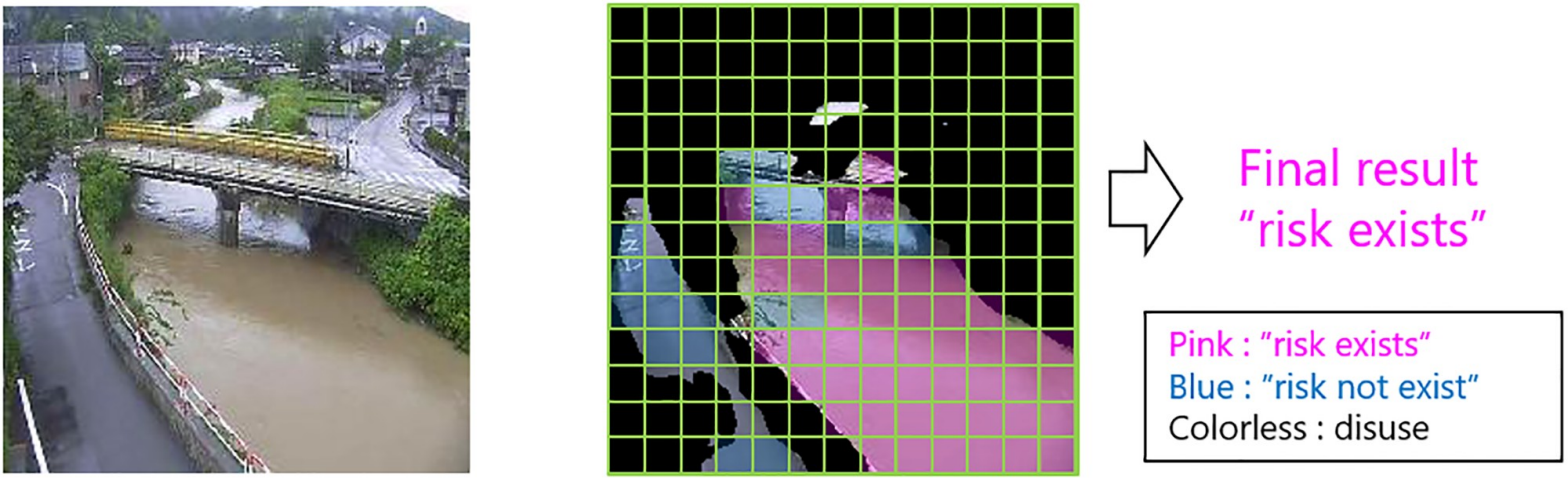
Example of the test phase using the trained model for river state classification.

Fig 3 shows an example of the testing phase of the proposed method. The middle of the figure illustrates image segmentation and patch-based data augmentation. The figure shows that these processes enable data augmentation when selecting relevant regions for classification. However, some irrelevant regions remain (such as the road regions in the bottom left of the image). Patch-based ensemble learning —that is, majority voting of the patch-wise test results— reduces the influence of the irrelevant regions, to realize accurate classification.

## Experimental results

Experimental results with real river images are presented to verify the effectiveness of the proposed method. As described above, the purpose of this work is the development of patch-based processing to adjust general CNNs to the river state classification. We verify that our proposed processing satisfies this purpose below.

### Dataset

The images were obtained from a dataset used for the previous studies [13, 28] and a dataset containing images from the river surveillance camera in Nagaoka City, Japan. The images in the former dataset are available from the database at http://places2.csail.mit.edu/index.html. We used images with a river label from this database. The images in the latter dataset are available from https://www.kishou.city.nagaoka.niigata.jp/camera/guest/. When flood risk increases, the color of the river appears to be brownish. Therefore, for the former dataset [13], we defined the river images with *Muddy* and *Clear* labels as positive and negative samples, respectively. The Muddy and Clear labels were defined in the previous study [13]. For the latter dataset, we defined the river images captured when the government announced heavy rain or flood warning as positive samples and other images as negative samples. The sizes of all images were 224 pixels × 224 pixels. We set the patch size and slide width as *C* = 32 and *P* = 16, respectively. Thus, by Eq (1), 169 patches were generated from each image. For training images, we performed contrast normalization [29] and mirroring [14]. Contrast normalization comprised both subtractive normalization and divisive normalization. Subtractive normalization is a process that subtracts the mean of all pixel values, of training data, from each pixel value [29]. This process normalizes the brightness of the input images, but may enhance noise. To tackle this problem, we also performed divisive normalization [29], which divides the results of subtractive normalization by the standard deviation of all pixel values of the training data.

### Experimental conditions

In this experiment, we verify the effectiveness of the proposed method by applying our method to recently proposed CNNs [14–20] (see Table 2). We used ResNet models whis 20, 50, and 101 layers. The widening parameter for WideResNet, *k*, was set to 4. In the training of the CNN model, we trained a model by solving the optimization problem using binary cross entropy as the loss function. The loss function *L* is defined as follows:

$$\begin{array}{c} L=-t\;\text{log}\;y-(1-t)\;\text{log}\;(1-y), \end{array}$$ (2)

where *t* is the ground truth class and *y* is the classification result from a model. For the solver of the optimization problem based on the loss function, we used stochastic gradient descent with momentum (momentum SGD) [30]. Momentum SGD [30] is an improved version of the SGD [31] algorithm, one of the most popular optimization algorithms used in deep learning. We set momentum and weight decay [32] to 0.9 and 0.0005, respectively. Here, weight decay is a scheme for avoiding overfitting caused by an excessive increase in weights. The number of epochs was set to 100. For AlexNet [14], NIN [15] VGGNet [17], and ResNet [18], we set the learning rate to 0.001, 0.001, 0.05, and 0.01, respectively; for other CNN models, we set the learning rate to 0.1. We multiplied the learning rate by 0.1 when the epoch number became 50. In addition, the mini-batch size was 32, and the initial values for the convolution filter were set as in [33]. Other parameters, such as the number of filters, were set in the same manner as in the original references.

**Table 2:** Number of layers used in CNNs.

CNNs	Number of layers
AlexNet [14]	8
NIN [15]	6
GoogLeNet [16]	22
VGGNet [17]	19
ResNet [18]	20/50/101
WideResNet [19]	16
ResNeXt [20]	50

### Parameter setting

**Fig 4:**
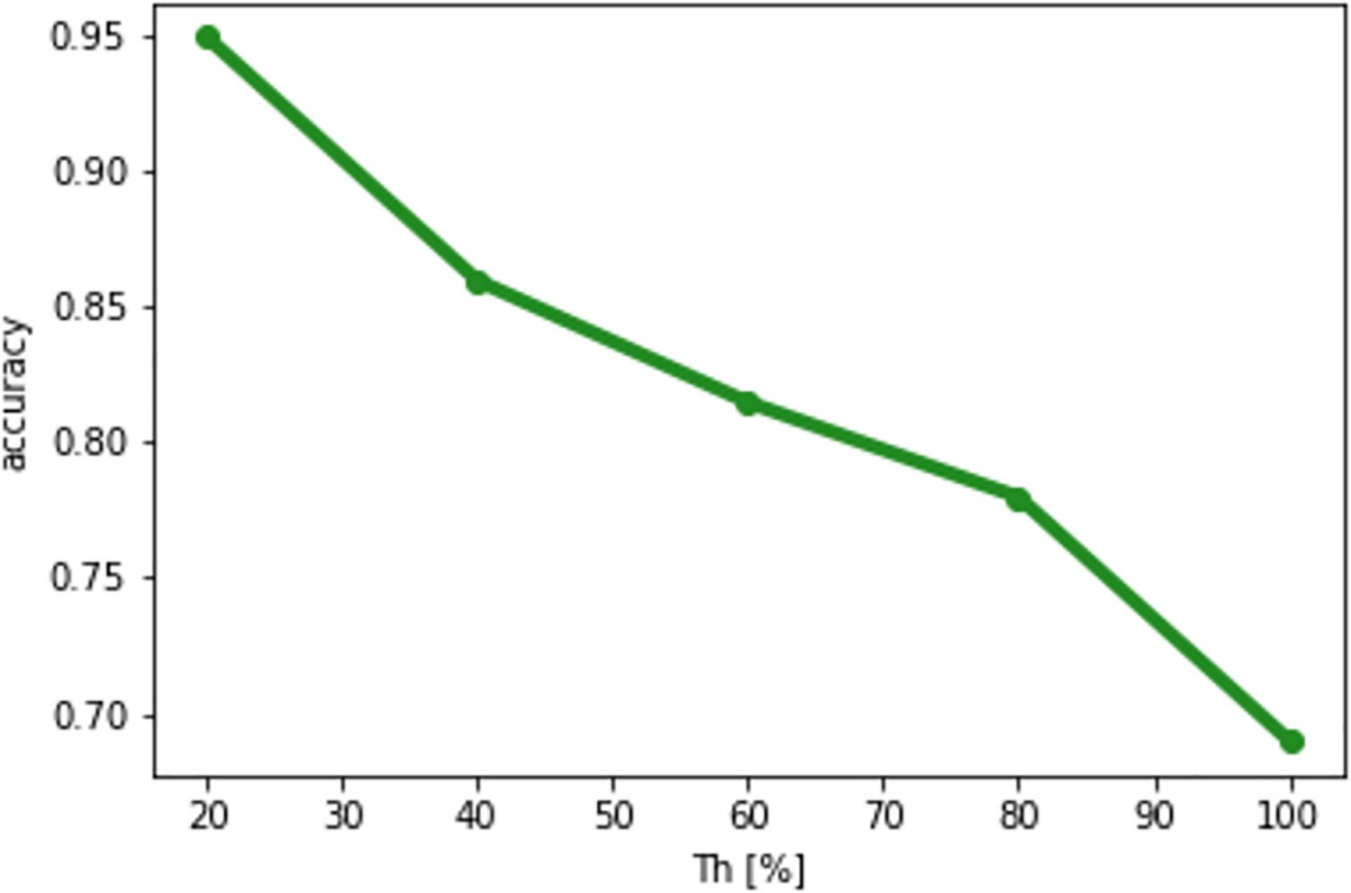
Relations between *Th* and the classification accuracy.

Here, we describe the setting of the parameter *Th*, which is used for selecting patches relevant to the classification. In this study, we validated the values *Th* = 20, 40, 60, 80, 100, which are expressed as percentages. We used 800 and 200 images for training and test, respectively. In this validation, we adopted AlexNet [14] as a CNN model. Fig 4 shows the result of validation; it shows that a more accurate result can be obtained with smaller value of *Th*. Therefore, we can conclude that *Th* should be small. If *Th* was set to less than 20%, all patches were removed, so that it was not possible to perform classification. Consequently, we defined *Th* as 20% for this experiment.

We describe a scheme for adjusting *Th* when we apply the proposed method to hundreds or thousands of river surveillance cameras. In such a situation, the suitable *Th* may be different depending on each photographing condition (e.g. places and the angle of view). Therefore, we should first collect images obtained in the similar conditions. Then, for each condition, we can adjust the suitable *Th* by dividing images into training and test images and validating the classification accuracy and *Th*.

### Results

For the evaluation, we performed five-fold cross validation [34] and calculated the following F-measure:

$$\begin{array}{c} F-measure=\frac{2\times Precision\times Recall}{Precision+Recall}, \end{array}$$ (3)

where

$$\begin{array}{c} Precision=\frac{TP}{TP+FP}, \end{array}$$

$$\begin{array}{c} Recall=\frac{TP}{TP+FN}. \end{array}$$

Here, TP is the number of samples that are correctly classified as positive samples. FP is the number of samples that are misclassified as positive samples. FN is the number of samples that are misclassified as negative samples. TN is the number of samples that are correctly classified as negative samples.

Table 3 shows the classification results for river surveillance camera images. Here, the columns show the results of our method (ours), the previous method [13] (Baseline 1), and the original CNN (Baseline 2). By comparing ours with Baseline 2, we confirmed that the adjustment of general CNNs to the river state classification became feasible by our pre-processing and post-processing. Note that Baseline 1 is a method that tries to adjust the general CNNs to the river state classification, but this method only incorporates image segmentation as pre-processing for CNNs. We confirmed that the proposed patch-based pre-processing and post-processing were superior than Baseline 1.

**Table 3:** F-measure scores.

	Average scores ± Standard deviations		
	Ours	Baseline 1 [13]	Baseline 2
AlexNet [14]	**0.887±0.089**	0.857±0.041	0.852±0.111
NIN [15]	**0.902±0.059**	0.854±0.040	0.738±0.086
GoogLeNet [16]	**0.928±0.043**	0.864±0.045	0.731±0.197
VGGNet [17]	0.903±0.043	**0.914±0.046**	0.717±0.120
ResNet20 [18]	**0.930±0.050**	0.772±0.114	0.728±0.046
ResNet50 [18]	**0.915±0.050**	0.818±0.073	0.734±0.060
ResNet101 [18]	**0.916±0.063**	0.734±0.059	0.648±0.156
WideResNet [19]	**0.946±0.043**	0.792±0.088	0.678±0.055
ResNeXt [20]	**0.913±0.063**	0.792±0.079	0.679±0.067
Average	**0.917±0.054**	0.824±0.065	0.726±0.09

**Fig 5:**
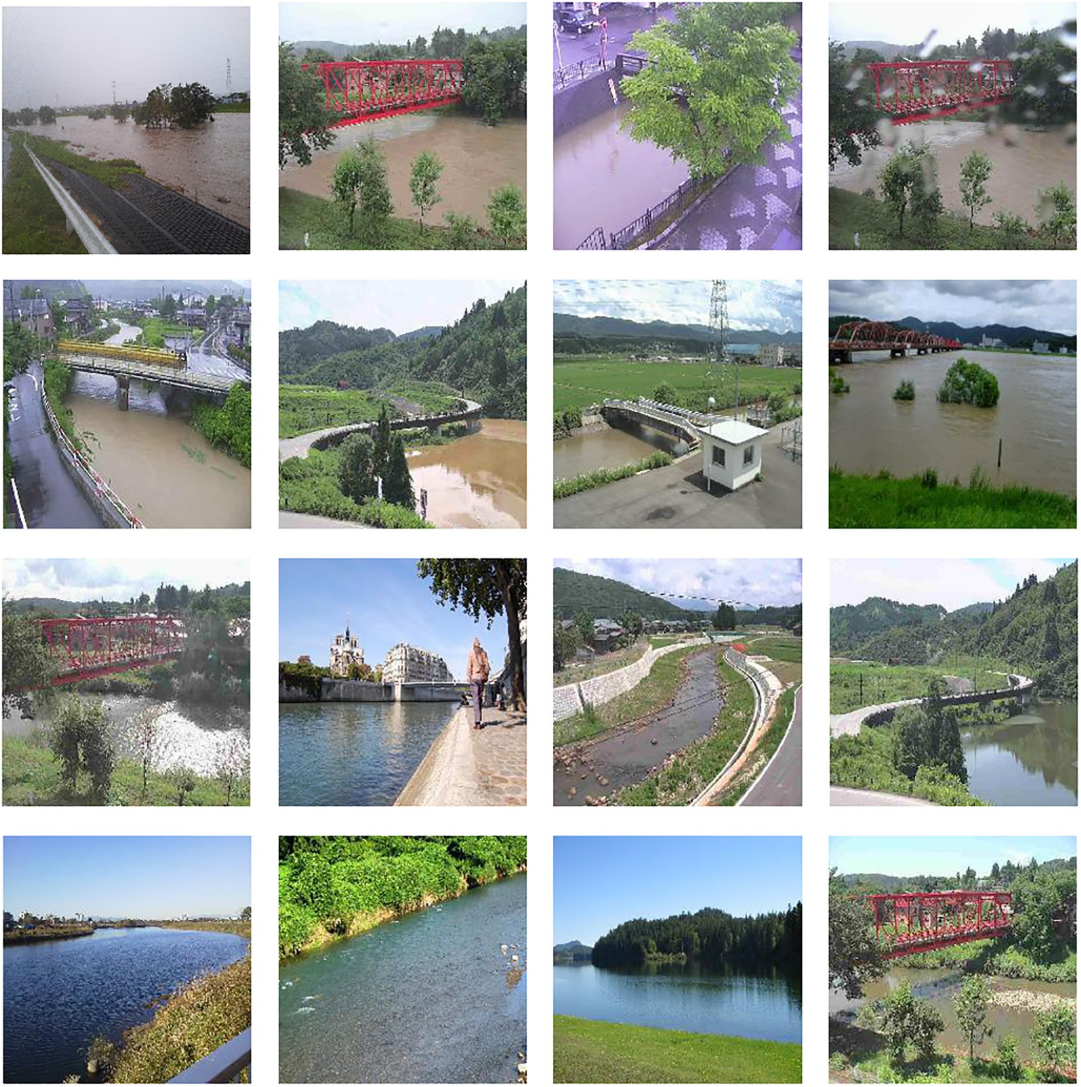
Examples of correct classification by our method. Two top rows: images when a flood risk exists; two bottom rows: images in the normal case (no flood risk).

For a detailed analysis, we performed Welch’s t-test [35] to the F-measures of ours and Baseline 1. As a result, we calculated a p-value of 0.000688, which statistically confirms the effectiveness of ours. Therefore, we can conclude that ours can accurately and robustly classify the river state. Fig 5 shows examples of classification results from ours with WideResNet. This figure shows that the appearance, such as color, varies between the images, according to the level of flood risk. Ours can suitably capture these differences in appearance, and perform successful classification.

### Discussion

We further discuss the effectiveness of our method in this section. From Table 3, it can be observed that our method increases the performance especially for WideResNet. This may be because this CNN model is suitable for small images. In fact, the original paper [19] shows that WideResNet is especially suitable for CIFAR10 [36], CIFAR100 [36] and street view house numbers (SVHN) [37], which include small images among many datasets. Also, for Baselines 1 and 2, Table 3 shows that the performance by ResNet101 [18] and ResNeXt [20] is low. This may be because these CNN models with many layers need much training data. Because ours can increase training data via the patch-based processing, we can successfully perform the river state classification even when using such CNN models.

**Fig 6:**
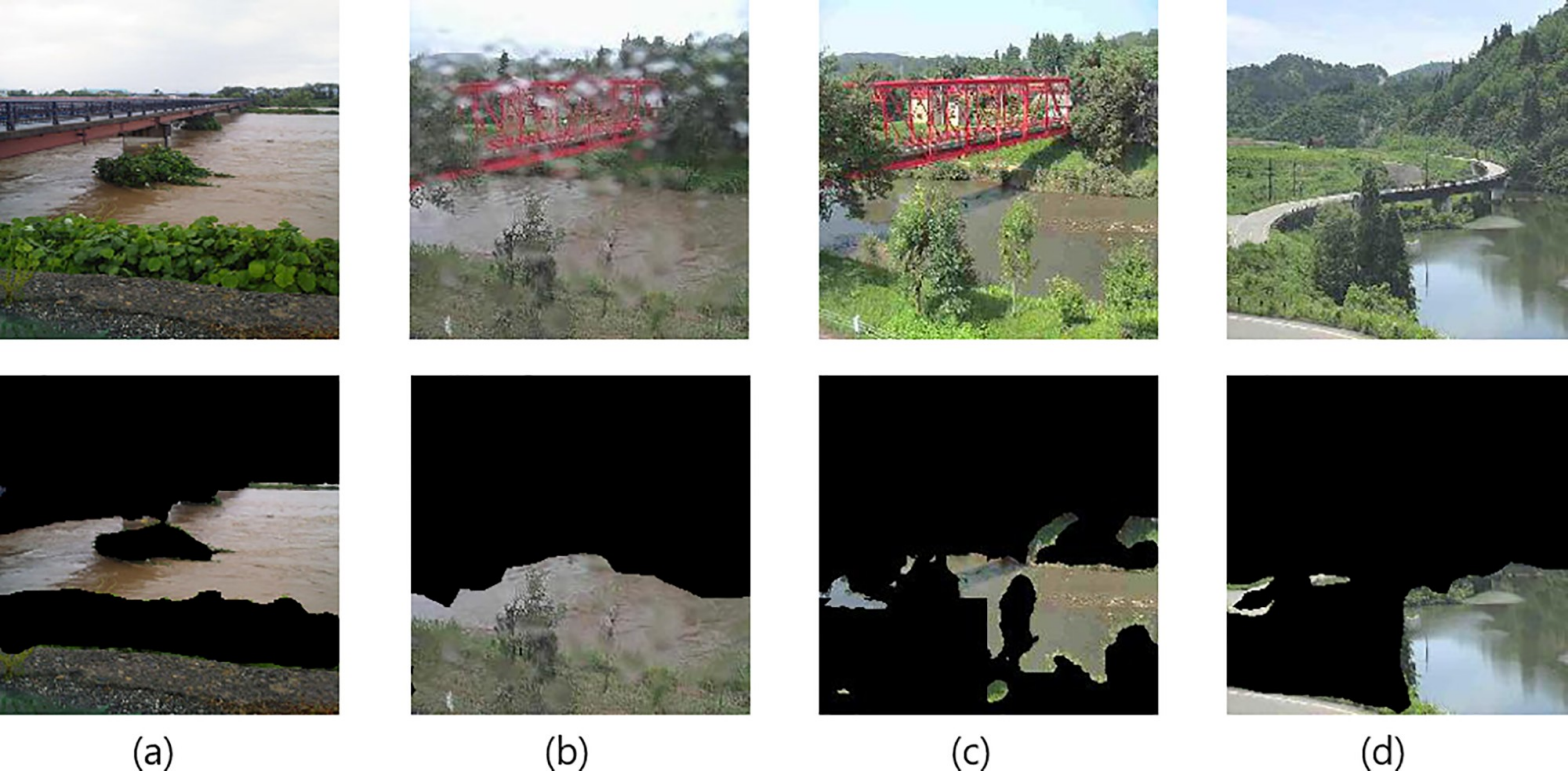
Examples of misclassification by Baseline 1 [13]. Upper row: original images; lower row: segmentation results.

Fig 6 shows examples that can be classified correctly by ours, but not by Baseline 1. Fig 6(b) and 6(c) show image of the same river. However, Fig 6(b) is a positive sample (flood risk case), whereas Fig 6(c) is a negative sample (normal case). Because the difference in appearance is small, these images may be difficult to classify correctly. However, ours can classify these images correctly because we can accurately train the river features by patch-based data augmentation in the training phase. For Fig 6(a), 6(b) and 6(d), Baseline 1 incorrectly detected some regions, such as the road and grassland as the river. This misclassification may occur because Baseline 1 trains and tests CNN models by using the incorrectly detected regions. In contrast, because ours performs ensemble learning —that is, patch selection in the training phase and majority voting in the test phase— accurate classification becomes feasible even if erroneously-detected water regions remain in the target image. As a result, we conclude that the adjustment of general CNNs to the river state classification becomes feasible by the proposed method.

## Conclusion

This paper proposed a method for classifying the river state (a flood risk exists or not) from river surveillance camera images by combining patch-based processing and CNN. Although CNN needs much training data, the number of river surveillance camera images is limited because flood does not frequently occur. Also, river surveillance camera images include objects that are irrelevant to the flood risk. Thus, the direct use of CNN may not work well for the river state classification. To overcome this limitation, we developed patch-based processing for adjusting CNN to the river state classification. By increasing training data and selecting relevant patches via the patch-based processing, the adjustment of general CNNs to the river state classification became feasible. We can develop the proposed patch-based processing and CNN independently. Therefore, we have the practical merits that any CNN can be used according to each user’s purposes, and the maintenance and improvement of each component of the whole system can be easily performed.

In the future, we will improve the ensemble algorithm by introducing boosting schemes such as [38, 39]. We are also interested in developing a new method for flood disaster prevention that can collaboratively use Web data such as weather information and Twitter posts as well as river surveillance camera images.
